# Apparent Young’s Modulus of the Adhesive in Numerical Modeling of Adhesive Joints

**DOI:** 10.3390/ma14020328

**Published:** 2021-01-11

**Authors:** Kamil Anasiewicz, Józef Kuczmaszewski

**Affiliations:** Department of Mechanical Engineering, Lublin University of Technology, 20-618 Lublin, Poland; j.kuczmaszewski@pollub.pl

**Keywords:** apparent Young’s modulus, adhesive joint modelling, adhesive joint zones, changes in adhesive properties

## Abstract

This article is an evaluation of the phenomena occurring in adhesive joints during curing and their consequences. Considering changes in the values of Young’s modulus distributed along the joint thickness, and potential changes in adhesive strength in the cured state, the use of a numerical model may make it possible to improve finite element simulation effects and bring their results closer to experimental data. The results of a tensile test of a double overlap adhesive joint sample, performed using an extensometer, are presented. This test allowed for the precise determination of the shear modulus *G* of the cured adhesive under experimental conditions. Then, on the basis of the research carried out so far, a numerical model was built, taking the differences observed in the properties of the joint material into account. The stress distribution in a three-zone adhesive joint was analyzed in comparison to the standard numerical model in which the adhesive in the joint was treated as isotropic. It is proposed that a joint model with three-zones, differing in the Young’s modulus values, is more accurate for mapping the experimental results.

## 1. Analysis of the State of Knowledge

The commercial range of adhesives from leading manufacturers includes a large selection of products dedicated to all industries. Adhesive manufacturers provide data on the physical properties of their products, e.g., bulk adhesive material and the strength of their adhesive bonds, as defined by available standards. These data are often sufficient to calculate the immediate strength of the adhesive joint made by the designer in complex machine elements, assuming a certain safety margin. Past research by the present authors has shown that the phenomena occurring in joints during the curing of the adhesive should be taken into account in order to increase the accuracy of calculations of the strength of the considered joint.

The design of modern machines and devices increasingly considers adhesive bonding as an indispensable technology of joining elements. Preparation of the adhered surface is of key importance due to the adhesive nature of the joint; however, the joint type and the material to be joined also have a decisive influence on the potential strength of the joint being prepared. Adhesive joints are difficult to evaluate in terms of modelling and strength calculations [1,2,3,4,5,6]. The complex physical and mechanical properties of adhesives, depending on the state of stress, strain, and temperature, need to be considered in order to deal with modelling issues. The geometrical structure of the joint, consisting of the joined elements and a very thin layer of adhesive between them, is important for the case in question [1,2,3,6]. According to existing research, the connector material, especially if it is metal, has a strong influence on the adhesive in the liquid state. As a result, the adhesive material in the joint acquires material properties different than those of the adhesive material when the joint is fully cured [7,8]. In the case of adhesive joints, determining the correct Young’s modulus or Poisson’s ratio of the cured adhesive may prove to be a complex problem, especially when it comes to bonding metal elements with the use of epoxy adhesives. The adhesive in the liquid state, in contact with the activated metal surface, is subject to physisorption and chemisorption forces at the interface. Surface activation is defined as the alteration of the surface chemistry by introducing chemical groups or charges, or introducing physical changes through etching, plasma plating, removing impurities by ablation, roughening, and void formation. Substance agglomeration on the surface of the adsorbent due to the action of intermolecular attraction forces is called physical adsorption. This results in a specific arrangement of the adhesive structure in the boundary zone, which has different properties compared to the central (core) part of the joint [1,4]. The specific arrangement refers to the ordering of molecules caused by the energetic influence on the adhesive layer being in direct contact with the metal surface [9]. Chemical adsorption involves the transfer, exchange, or sharing of electrons between adsorbates and adsorbents, and the adsorption of adsorbates on adsorbents is due to the formation of chemical bonds between them. Physical and chemical adsorption phenomena can occur simultaneously. These interactions, acting on epoxy resin particles, cause them to become concentrated [4]. The consequences of the presented phenomena are called the apparent Young’s modulus, *E_k_*. Within a certain range of applications and thicknesses of adhesive joints, these changes can cause errors in the design of adhesive joints and inaccuracies in the calculation of their strength. It is assumed that the strengthening of the boundary zone may have a significant impact on the strength of the joint in cases of joints of small thickness [1,8,10,11].

**Figure 1:**
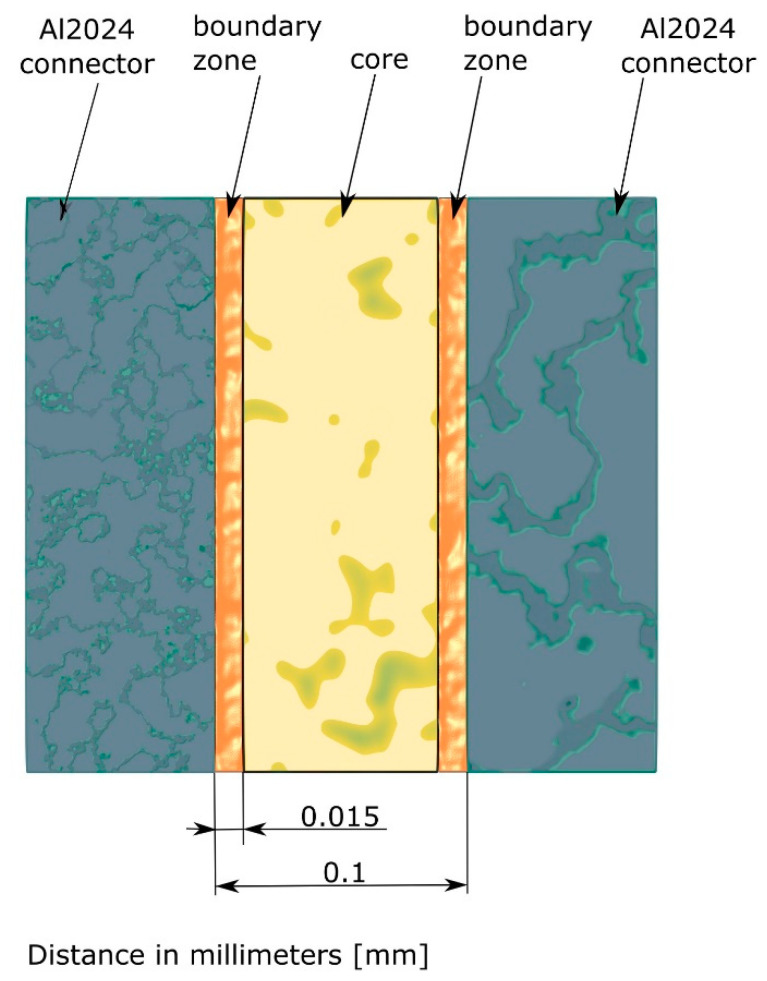
Schematic representation of adhesive joint with indicated zones.

Literature sources indicate differences in the measured values of the tensile strength of adhesive materials in terms of the shape of the bulk adhesive specimen, compared to the tensile strength of joints of different thickness in the butt joint. The gradual decline of these differences was also observed with the increase in the thickness of the adhesive joint [10,12]. Also, in the course of the present authors’ research, a decrease in the strength of the butt adhesive joint was observed with the increase in the thickness of the joint. This may have been due to the increased amount of defects and other factors compromising the cohesive strength of the joint that can be expected with larger adhesive joints. The observed reduction of joint strength, understood in this way, may have been caused by the lack of direct contact of the joint core with the joined material during the joint cross-linking, which resulted in the disorganization of monomer molecules. Another issue that should be discussed in terms of changes in the physical properties of the adhesive in the joint is the cross-linking of polymers. Cross-linking occurs by means of van der Waals interactions or covalent chemical bonds. The strength of these interactions is correlated with cross-link density. The high-density polymer network is characterized by a greater number of knots, which usually results in increased stiffness of the cured polymer material [1,13,14,15]. In joints with a thickness greater than 0.15 mm, this phenomenon is confined to the boundary zone, and its effect on the material properties gradually disappears. In the authors’ own research of the properties of thin adhesive layers, the presence of boundary zones with different material properties compared to the joint core was observed. The thickness and differences in the values of Young’s modulus and the hardness of individual layers was determined. The obtained results were then analyzed in terms of single-lap adhesive joints. Following nanoindentation tests, it was found that each boundary zone with an increased Young’s modulus reached up to 0.015 mm from the joint edge, which constituted, in total, up to 30% (for two boundary zones) or more of the total joint thickness, assuming that it was about 0.1 mm in thickness. The value of Young’s modulus in the boundary zone was 15% higher compared to the core value [7]. In the aforementioned research, no destructive tests were carried out and no direct comparison of the experimental results with the numerical simulation was made; as such, these notions are the subject of the present article. A schematic representation of an adhesive joint with zone division is shown in Figure 1.

Predicting the strength of adhesive joints in complex structures is complicated. Therefore, a more refined model of the adhesive joint can significantly affect the accuracy of modelling. It is all the more important to predict the behavior of an adhesive joint in terms of its elastic deformation, as it is in this range that the main part of the exploitation of adhesive joints occurs in the long term, specifically, in terms of long-term variable fatigue stress. Determining the influence of changes in the properties (stiffness) of the joint on the elastic behavior will have a decisive influence on the construction of a detailed model covering the physical-mechanical properties of adhesive joints [6,16,17].

The adhesive joint and test conditions considered in this paper do not involve complicated models which take into account temperature amplitudes and fatigue stresses. In the general modelling approach, depending on the problem under consideration, the following stress hypotheses are used: maximum failure stress, maximum failure strain, or the von Mises (HMH) yield criterion [3], modelled based on the characteristic σ = σ (ε). In popular numerical models, the failure criterion is often used, taking into account the traction-separation criterion (1), where *t_n_* is tensile force and *t_s_* is shear force. In the Abaqus program, the application of the aforementioned criterion is possible thanks to cohesive elements in the so-called CZM (cohesive zone model) [2].

(1) $${\left\{\frac{\langle t_{n}\rangle }{t_{n}^{0}}\right\}}^{2}+\;{\left\{\frac{t_{s}}{t_{s}^{0}}\right\}}^{2}=1$$

Simulations which consider the presented criteria usually correspond well to experimental tests of adhesive joints, but rely on experimentally determined input data. These models do not take into account the division of the adhesive joint into three layers of different values of Young modulus, according to the authors’ research [7].

## 2. Experimental Studies

**Figure 2:**
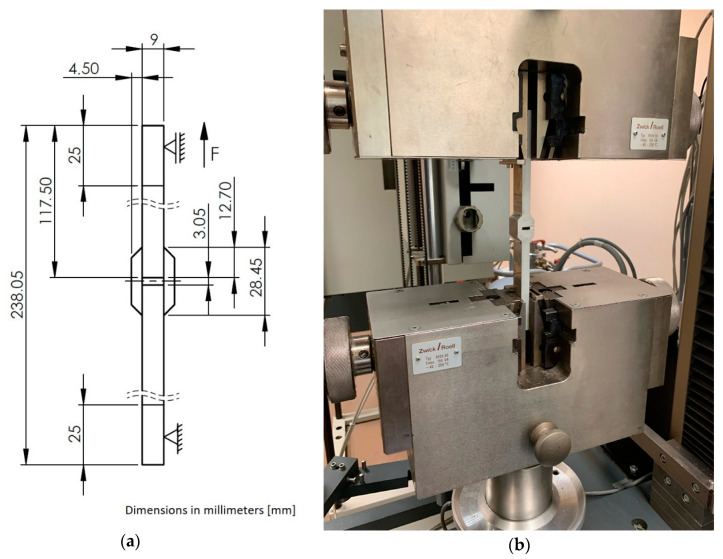
The drawing of a 25.4 mm wide double-lap sample with the main dimensions (**a**). An example of a sample compliant with ASTM 3528 in the test rig of a Zwick Roell Z150 testing machine (**b**).

The aim of our research was to develop and experimentally verify a new approach to the modelling of an adhesive joint, which takes changed adhesive material properties of the joint into account. Adhesive material properties cured in adhesive joints differ in comparison to bulk adhesive material properties. In order to verify the presented assumptions, double-lap samples were made in accordance with the ASTM D3528 standard [18]. The samples were made in the form of panels which, after joining, were cut into individual samples. Connectors and laps were made of AW 2024-T3 aluminum alloy. The surface of connectors and laps was cleaned with Loctite SF 7063 universal cleaner using a cloth, then brushed with a P320-grade, nonwoven fabric and cleaned again with a universal cleaner. The surface of the connectors and laps prepared in this way was plasma plated. Plasma plating was carried out in three head passages at 150 W, helium flow 30 L/min, and oxygen 0.3 L/min. The elements were joined with Loctite Hysol EA 9392 Aero adhesive, which is a two-component adhesive for joints used in the aviation industry. It is characterized by its high strength within a large temperature range, to which adhesive joints used in aviation structures are often subjected. The adhesive used is intended for joining metal, composites, and honeycomb elements. The adhesive is used by most of the leading part manufacturers in the aviation industry. The adhesive was prepared in proportion to weight, in accordance with the manufacturer’s recommendations, and evenly distributed on the adhesive surfaces using a special spatula. The lap was positioned in relation to the connectors on the fixing pins. The joint was cured for 24 h at room temperature with pressure in a vacuum sleeve, under a pressure of 0.1 MPa. After seven days, the panels were cut with a waterjet into individual samples with a width specified in the standard—25.4 mm. The samples were cleaned of the adhesive flash to maintain the lap length specified in the standard. All relevant dimensions were checked and the thickness of the adhesive bond was measured before starting the test. Joint thickness was measured with a Keyence VHX-5000 digital microscope. The average bond thickness of ten samples was 0.102 mm with a coefficient of variation of 1.72%. Two strength tests were carried out at room temperature. In the first test, five samples were tensile tested with the use of a traverse. In this way, the average destructive force of the adhesive joint was determined. The second tensile test was carried out with an extensometer which was compliant with the standard PN-EN ISO 9513: 2013-06. The samples were stressed up to 70% of the destructive stress value, determining the stress-strain characteristics for the considered joint. Five repetitions were performed for each series. A drawing of the sample with its dimensions and boundary conditions (a), and a photograph of the sample in the test rig of the Zwick Roell Z150 testing machine (b), are shown in Figure 2.

The obtained results were applied to a numerical model. The numerical model was built as a 2D model in the Abaqus environment with a geometry that was identical to those of the samples used in a previous study [19]. The material of the connectors and laps was AW 2024-T3 aluminum alloy. Each lap length was 12.7 mm. The adhesive material in the first model, designated as “single-zone”, was determined following the catalogue cards provided by the adhesive manufacturer, according to well-known standards followed in the aviation industry [20,21]. A numerical elastic-plastic model was used for the adhesive material. The joints between the adhesive surfaces and the connectors were defined by surface-to-surface contact, and ties in between were set as “Tie” which resulted in all nodes of joint elements becoming attached to the adherend surface.

The individual parts of the adhesive joint, including laps, connectors and joints, are based on a structural mesh of volumetric hexagonal finite elements. The solids were divided into finite elements of the “Plane stress” type, appropriate for modelling thin geometries as well as representing a plane state of stress. The densities of the finite elements along the thickness of the adhesive joint were 70/mm for the one-zone model and 100/mm for the three-zone model, with the latter being thicker due to the presence of additional zones. CPE4R finite elements were used to mesh the joint. A static tensile test was simulated. The developed numerical representation of the double-lap joint was intended to achieve the best possible representation of the tensile test of the joint. The geometries of the connectors, laps, and joint were developed with actual dimensions and their mutual arrangement. Deformations of connectors and laps was taken into account. The adhesive joint material had elastic-plastic properties defined by the stress-strain characteristics σ = σ(ε), obtained experimentally. A flat deformation condition prevailed in the adhesive joint [1].

**Figure 3:**
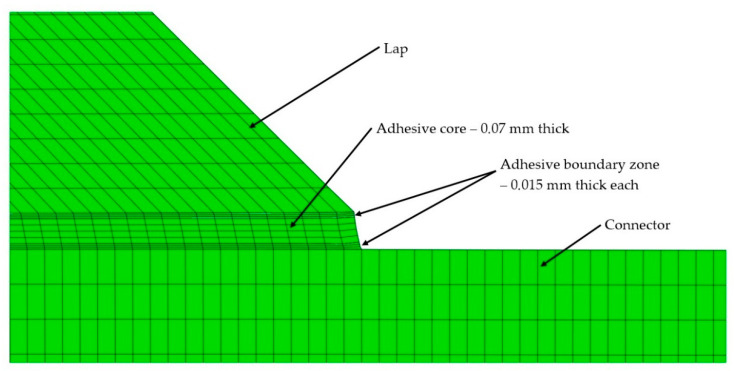
A detailed view of the numerical model with the presented division of the joint into three zones, with visible mesh density in the boundary zone.

The second numerical model was prepared similarly to the first one, but with the difference that the joint was modelled as a three-zone joint, and included the phenomena that accompany the curing of the adhesive during the formation of the adhesive joint, based on the authors’ own research [7]. Three zones were identified in the adhesive material, dividing the adhesive joint model into three separate areas. Two boundary zones and the core of the adhesive joint were designated. Based on past research by the present authors’, the two boundary zones which were in direct contact with the surface of the combined material were assigned the value of the apparent Young’s modulus, i.e., 15% higher (based on nanoindentation tests) in relation to the basic value of the adhesive material. The inclusion of a three-zone boundary layer in the model with a thickness of 0.015 mm determined the maximum size of a single finite element in the layer thickness as 0.005 mm. This was dictated by the necessity to discretize the model into at least three finite elements. The material properties of the adhesive were adopted on the basis of the manufacturer’s specifications, in accordance with data presented in Table 1. The division of the adhesive joint into three layers is shown in Figure 3. The HMH criterion for plane state of stress was used in the simulation. In order to reproduce the conditions of the experimental test with an extensometer whose distance between the caliper arms was set to 50 mm in the model, the distance between the attachment points and the force application was also defined as 50 mm. This allowed a direct comparison to be made between the experimentally obtained results and the simulation results in the studied range of force impact.

The force equal to 70% of the destructive force value obtained in the first experimental tensile test was used as the driving element in the simulation. The force applied to the sample in the simulation was F = 11,480 N. In order to compare the obtained experimental and numerical results, the stress-strain curve and the stress distribution along the lap for the modelled time step were generated in the Abaqus program. The obtained results were developed for both models.

## 3. Results

**Figure 4:**
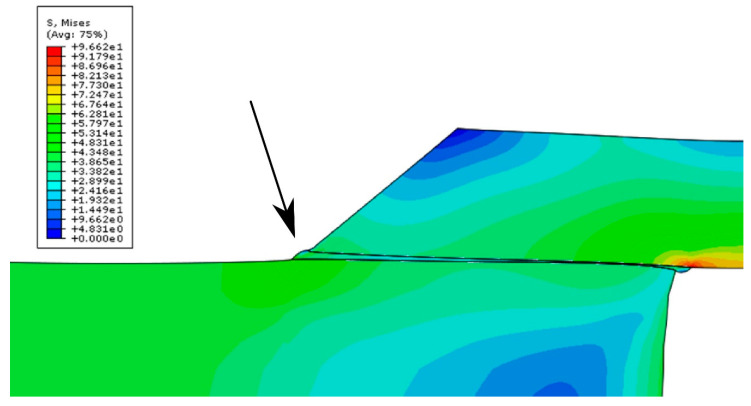
Stress bitmap shown on the 1/4 part of the FEM model. Representation of displacements with a deformation factor ×150.

The bitmap in Figure 4 presents a visualization of the stress distribution in a double-lap joint. Because stresses were symmetrically distributed in all four joints of the double overlap sample, one-fourth of the model was presented. The deformation coefficient was increased to ×150 in order to illustrate the deformation of the adhesive joint. The image shows the deformation of the joint due to tension at the end of the lap. The end of the lap is indicated since it was the region where destruction of the adhesive joint was initiated by normal stresses in the adhesive joint that arose during the shearing of the joint. Tension along the thickness of the joint can be observed. It is important to mention that the Young’s modulus of the adhesive influences the potential for the deformation of laps and connectors of the adhesive connection.

**Figure 5:**
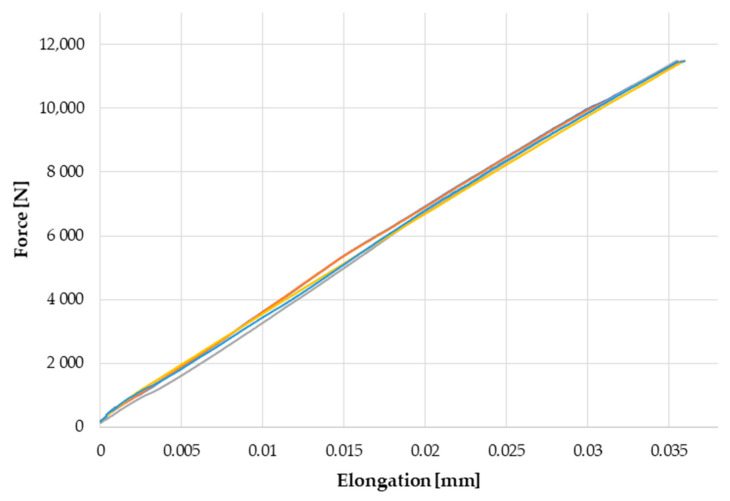
Experimental results of tension test of double overlap sample according to ASTM D3528.

**Figure 6:**
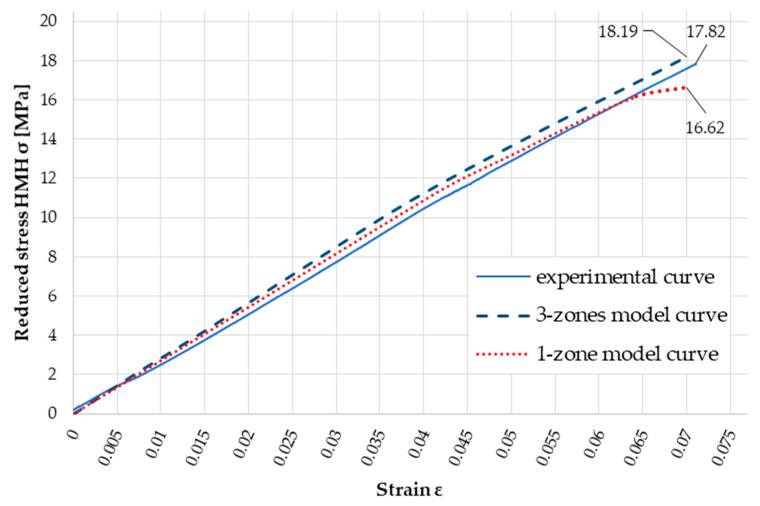
Comparison of the experimentally obtained stress-strain curve and the results obtained from the FEM simulation.

The graph shown in Figure 5 presents the experimental results of five tension tests. The experimental curve represents the average of five samples that underwent tension tests with variance %RSD of 3.1%. Considering maximum elongation at maximum applied force variance %RSD was 0.6%. The high accuracy of the results was due to the preparation of samples which were initially bonded as one panel. The simulation results of the one- and three-zone models compared with the experimental results are shown in Figure 6. The experimental curve represents the average of five tests. In the attached graph, a high degree of coincidence between the experimental curves and those obtained as a result of the simulation is visible. The curve representing the one-zone model is worth noting. Starting with a relative equal deformation of 0.055, a slope in this curve which is characteristic of the beginning of the plastic deformation can be observed. The presented curve should be considered in relation to the physical properties of the adhesive material, which, due to the lack of strengthening, was characterized by a lower average value of the Young’s modulus in relation to the adhesive joint, and thus, lower stiffness. Such a curvature may indicate premature failure of the joint. A higher coincidence was observed with the results obtained experimentally in the case of the chart representing the three-zone model.

It was confirmed that the computational characteristics fit well with the experimental data. Therefore, computer FEM simulations using the three-zone joint model return more accurate outcomes than the one-zone model. This conformity was achieved by considering the detailed results of the variable distribution of the Young’s modulus values on the thickness of the adhesive joint, the physical properties of which differ from those of the structural material in the form of a dumbbell cast sample.

**Figure 7:**
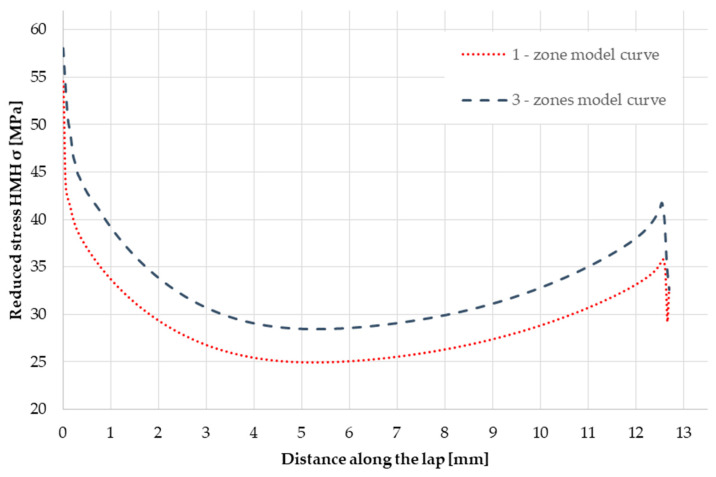
Comparison of reduced stress along a single 12.7-mm long lap.

Figure 7 presents simulation curves of reduced stresses along the length of a model lap, i.e., along the dimension 12.7 mm. Nodes that were in direct contact with the connector material were selected for comparison. In this case, the difference in stress at the end of the lap was observed, which affected the initiation of joint destruction. Due to the analogies between the construction of the numerical one- and three-zone models, a similar shape of the curves was observed. The results can also be analyzed in terms of the subsequent destruction of the adhesive joint, which was often of an adhesive or adhesive-cohesive nature. Higher stresses in the boundary zone were directly related to the destruction of the joint and the difference in the material properties of the adhesive in the joint core and the boundary zone; consequently, a different stress state in these areas may be considered as one of the reasons for the observed destruction.

## 4. Summary

The main aim of our research was to confirm the merits of including changed zones of joints with different properties in relation to the core material used in the simulation. A comparison of the FEM simulation results with the actual results obtained in the axial tensile test confirmed the need to consider the changed boundary zone in FEM modelling in order to improve accuracy. The presented results apply to a specific type of adhesive; therefore, the presented conclusions should be considered as being representative of a specific case.

The placement of boundary zones in the construction of the adhesive joint model allowed for a higher compliance of the sigma/epsilon curve, compared to the model with a single-zone joint. The discrepancy in the maximum stresses at the same strain level for the one-zone model was 6.7%, while for the three-zone model, the value of the maximum stresses differed from the experimental stresses by only 2.1%. It should also be noted that the displacement obtained in the simulation resulted in stresses only in the elastic range of the adhesive material. The model that included three layers of adhesive was at a 95% confidence level of the experimental values. The main conclusions are as follows:

1. In this comparative study, the experimental results were found to be consistent with the FEM simulation results.
2. The experimental tests of a double-lap sample according to ASTM D3528 were characterized by high repeatability, with variance of <1%.
3. As part of comparing the experimental results with the simulation results of a connection with an isotropic (one-zone) joint, the graph showed the consistency of the course in the initial part and a noticeable beginning of plastic deformation in the last part. For the simulation of a joint that included boundary zones, an equally high degree of similarity was observed in the first part of the graph, with the difference that at the end of the course, the graph had a higher degree of compliance with the course of the experimentally obtained curve.
4. It can be concluded that the three-zone joint model more accurately reflected the stress-strain curve for the considered double-lap sample. The observed compliance may confirm the influence of the cured part of the joint on the joint stiffness.
5. The use of the FEM model—which takes additional boundary zones of the adhesive joint into account—influenced the reduction of the achieved stresses according to the HMH hypothesis. In the area of the end of the lap, the stress was 7.4% higher in the model including three zones in the adhesive joint compared to the traditional model which treated the entire volume of adhesive as isotropic.

Adhesive joint decohesion usually begins in the boundary zone. Hence, it is important to determine the material constants as precisely as possible for simulations. The scientific value of the present work is, inter alia, the indication that after exceeding a certain load, the differences between the stress determined in the destructive test and that resulting from the numerical simulation are clearly visible. In terms of determining stresses close to failure load, the three-zone model is more appropriate.

Increased stresses at the ends of the adhesive joint lap have a decisive influence on the strength of double overlap joints, because it is the normal stresses at the ends of the lap that most often initiate the destruction of the joint. Considering the changes occurring in the adhesive joint during curing allows more accurate predictions to be made of the joint strength and operation. 

## Figures and Tables

**Table 1:** Material properties of materials used in the FEM model.

Model Body.	Connector and Lap	Adhesive Core	Adhesive Boundary Zone
Material	AW 2024-T3	Loctite Hysol EA 9392 Aero	Loctite Hysol EA 9392 Aero
Young’s modulus [GPa]	73,000 MPa	3273 MPa	3770 MPa
Poisson ratio [-]	0.33	0.34	0.34
Tensile strength [MPa]	430 MPa	41.3 MPa	41.3 MPa
Extension at break [%]	15	4	4

## Data Availability

The data presented in this study are available on request from the corresponding author.
